# Tissue specific expression of extracellular microRNA in human breast cancers and normal human breast tissue *in vivo*

**DOI:** 10.18632/oncotarget.4038

**Published:** 2015-05-08

**Authors:** Annelie Abrahamsson, Charlotta Dabrosin

**Affiliations:** ^1^ Department of Oncology and Department of Clinical and Experimental Medicine, Linköping University, Linköping, Sweden

**Keywords:** mammary gland, microdialysis, sex steroids, estrogen, tamoxifen

## Abstract

Extracellular circulating microRNAs (miRNAs) have been suggested to be biomarkers for disease monitoring but data are inconsistent, one reason being that blood miRNA is of heterogeneous origin. Here, we sampled extracellular microRNAs locally *in situ* using microdialysis. Three different cohorts of women were included; postmenopausal women with ongoing breast cancer investigated within the cancer and in normal adjacent breast tissue, postmenopausal women investigated in their normal healthy breast and subcutaneous fat before and after six weeks of tamoxifen therapy, premenopausal women during the menstrual cycle. Samples were initially screened using TaqMan array cards with subsequently absolute quantification. 124 miRNA were expressed in microdialysates. After absolute quantifications extracellular miRNA-21 was found to be significantly increased in breast cancer. In addition, the levels were significantly higher in pre-menopausal breast tissue compared with postmenopausal. In breast tissue of pre-menopausal women miRNA-21 exhibited a cyclic variation during the menstrual cycle and in postmenopausal women six weeks of tamoxifen treatment decreased miRNA-21 suggesting that this miRNA may be important for breast carcinogenesis. None of these changes were found in plasma or microdialysates from subcutaneous fat. Our data revealed tissue specific changes of extracellular circulating miRNAs that would be otherwise unraveled using blood samples.

## INTRODUCTION

MicroRNAs (miRNAs) are small endogenous non-coding molecules of approximately 21-25 nucleotides that regulate transcription of numerous genes by binding to complementary sequences on the 3′un-translated regions (3′UTR) of the messenger RNA molecule [1]. De-regulation of miRNAs has been associated by onset and progression of various pathological processes including breast cancer [2]. Since the discovery of circulating miRNAs in different body fluids it has been suggested that miRNAs not only act within the cells, but are also exported into the extracellular space [3, 4]. These extracellular circulating miRNAs has been shown to be transported in membrane bound vesicles or in different protein-complexes including nucleophosmin 1 (NPM1) and argonaute 2 protein (Ago 2) [4–6]. It has been suggested that circulating miRNAs in serum or plasma may serve as useful biomarkers of disease progression as previous studies have shown a correlation between blood miRNAs and stage of cancer [3, 7, 8]. However, there is a lack of consistency between different studies and cohorts. There may be several explanations of this including a lack of standardized protocols for sample preparation and normalizations procedures. In addition, for extracellular miRNAs no individual or sets of miRNAs have been validated to be invariant and suitable as endogenous controls [9]. As there are no specific endogenous control for extracellular miRNAs a fixed volume rather than a fixed amount of RNA should be used as an input as recently described [9].

Extracellular miRNAs may be expressed tissue specific and function as genetic communicators between cells and thereby affecting the microenvironment in specifying and maintaining tissue identity [10–12]. Thus, one major problem in using serum or plasma miRNAs as biomarkers is that these are of heterogeneous origin and will reflect the net amount of the total extracellular miRNA in the body rather than extracellular miRNA from a specific tissue.

Hitherto, to our knowledge, no previous data of extracellular miRNAs sampled from live human tissues has been reported.

The aims of this study were to explore if microdialysis, which previously has been shown to be a viable technique for sampling of extracellular compounds in human breast tissue [13–18], could be used for sampling of extracellular miRNAs in human breast cancers and normal breast tissue *in vivo* and if any differences in expression levels of specific miRNAs could be found in these tissues. We also investigated if the transport proteins NPM1 and Ago 2 passed the microdialysis membrane. We show that extracellular miRNAs in microdialysis samples are stable and exhibit very low extraction variability and that the transport proteins pass the microdialysis membrane. 124 miRNA were expressed in microdialysates of human breast cancer. Our most significant finding was miRNA-21, which was significantly up-regulated in breast cancers and increased in normal human breast tissue during high levels of sex steroids and down-regulated by treatment with the anti-estrogen tamoxifen. None of these findings were detected in plasma or microdialysates from subcutaneous abdominal fat emphasizing the need of tissue specific sampling of extracellular miRNAs.

## RESULTS

### Tissue specific levels of extracellular miRNAs in breast cancer and normal breast tissue

As no previous studies have been published of miRNA expression in extracellular microdialysates we first explored if any miRNAs were detectable the samples. For this screening we used a human miRNA card of 377 different miRNAs. Microdialysis samples from five breast cancer patients were analyzed. Out of the 377 miRNAs on the card, 124 were expressed in the samples i.e. exhibited a CT-value less than 35, Supplementary Table S1.

Out of these 124, 9 miRNAs previously reported to be involved in cancer progression and/or were readily detectable in the microdialysis samples during the screening process were chosen for absolute quantification using real-time PCR with serial diluted synthetic miRNAs as standard curves. Standard curves of the selected miRNAs are shown in Supplementary Figure S1. The majority of these 9 miRNAs has previously only been detected in tissue specimen, representing intracellular miRNA rather than circulating miRNAs, whereas some also have been detected in blood samples [19–28].

**Figure 1:**
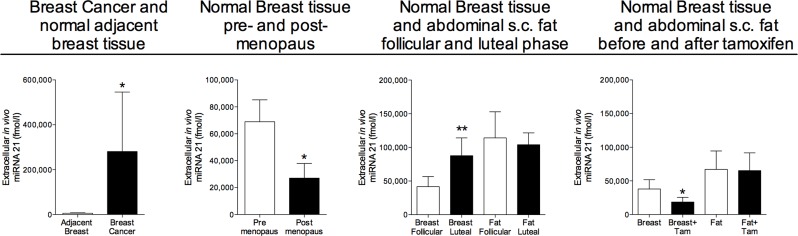
Increased extracellular *in vivo* levels of miRNA-21 in human breast cancers and normal human breast tissue of women during increased sex steroid levels Microdialysis was used for sampling of extracellular miRNA-21 *in vivo* in breast cancer and normal adjacent breast tissue in women before surgery, *n* = 13, in normal human breast tissue in pre-menopausal women, *n* = 16, and postmenopausal women, *n* = 12, in normal human breast tissue and abdominal subcutaneous fat of pre-menopausal women during the follicular and luteal phases of one menstrual cycle, *n* = 8, and in normal human breast tissue and abdominal subcutaneous fat of postmenopausal women before and after six weeks of tamoxifen therapy, *n* = 12. Bars represent mean±SEM, *p < 0.05, **p < 0.01.

As shown in Figure 1 miRNA-21 was significantly increased in breast cancers compared with normal adjacent breast tissue. In addition to the changes in breast cancers, miRNA-21 also exhibited changes in normal breast tissue during various hormone exposures. The levels of miRNA-21 was significantly decreased in normal breast tissue from postmenopausal women compared to pre-menopausal, Figure 1. In pre-menopausal women, the levels of miRNA-21 increased in the lutheal phase of the menstrual cycle when the levels of estradiol and progesterone are increased. In postmenopausal women the levels significantly decreased after six weeks of tamoxifen therapy, Figure 1. No significantly changed levels were seen in microdialysates from abdominal subcutaneous fat during hormone exposures, Figure 1.

As previously shown in plasma from both humans and mice, there was a wide inter-individual range of the absolute levels of all miRNAs in plasma [29]. miRNA-21 did not exhibit any significant changes during various hormone exposures (numbers in mean±SEM); 20271±6142 fmol/l in unexposed postmenopausal women compared to 180414±106090 fmol/l in pre-menopausal women, 122512±81997 fmol/l in the follicular phase *vs*. 238316±201381 fmol /l in the luteal phase and 20271±6142 fmol/l before tamoxifen therapy *vs*. 14451±1908 fmol/l after tamoxifen for six weeks.

**Figure 2:**
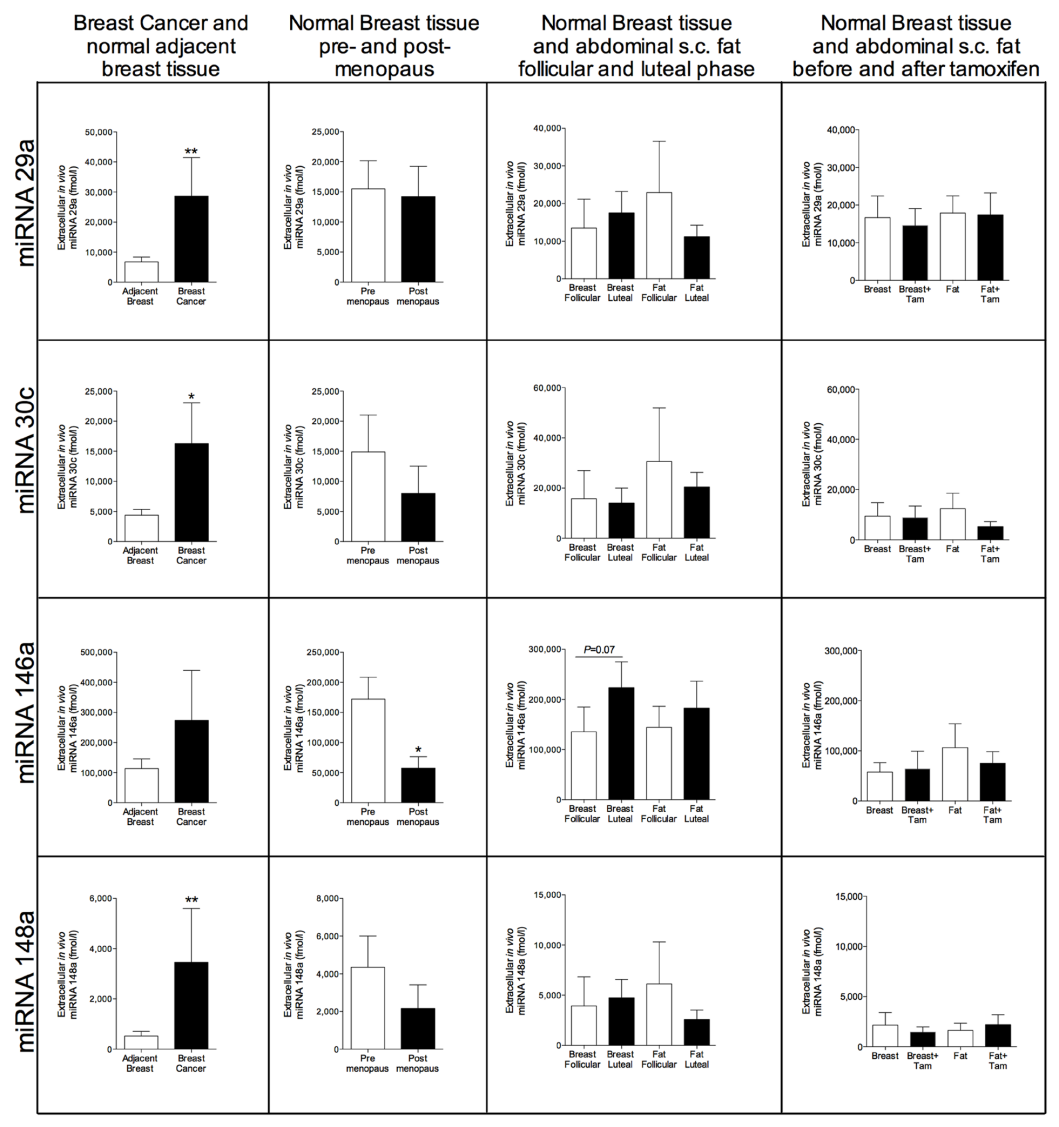
Extracellular *in vivo* levels of miRNAs in breast cancers and normal breast tissue of women during various hormone exposures Microdialysis was used for sampling of extracellular miRNAs *in vivo* in breast cancer and normal adjacent breast tissue in women before surgery, *n* = 13, in normal human breast tissue in pre-menopausal women, *n* = 16, and postmenopausal women, *n* = 12, in normal human breast tissue and abdominal subcutaneous fat of pre-menopausal women during the follicular and luteal phases of one menstrual cycle, *n* = 8, and in normal human breast tissue and abdominal subcutaneous fat of postmenopausal women before and after six weeks of tamoxifen therapy, *n* = 12. Bars represent mean±SEM, *p < 0.05, **p < 0.01.

**Figure 3:**
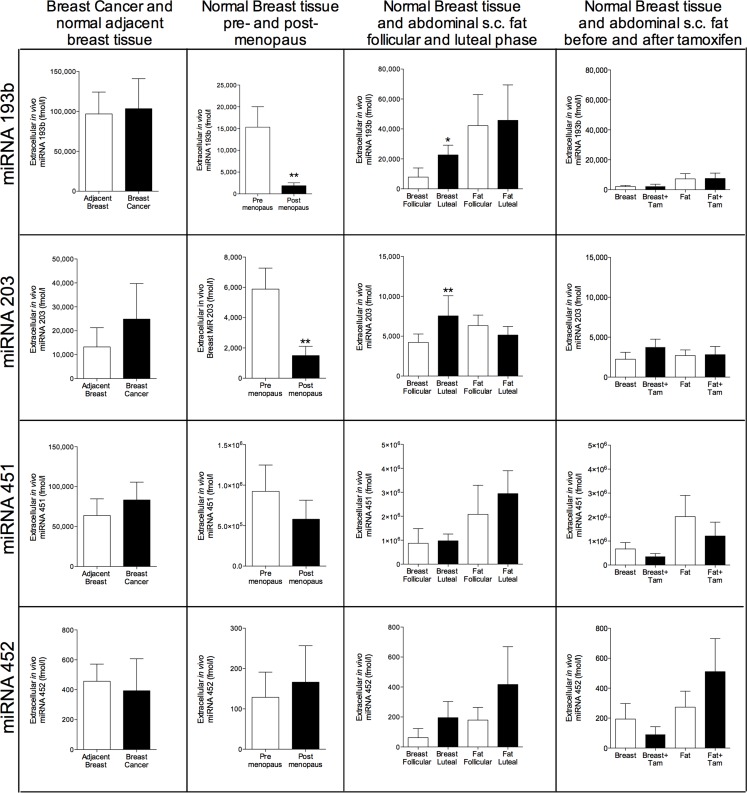
Extracellular *in vivo* levels of miRNAs in breast cancers and normal breast tissue of women during various hormone exposures Microdialysis was used for sampling of extracellular miRNAs *in vivo* in breast cancer and normal adjacent breast tissue in women before surgery, *n* = 13, in normal human breast tissue in pre-menopausal women, *n* = 16, and postmenopausal women, *n* = 12, in normal human breast tissue and abdominal subcutaneous fat of pre-menopausal women during the follicular and luteal phases of one menstrual cycle, *n* = 8, and in normal human breast tissue and abdominal subcutaneous fat of postmenopausal women before and after six weeks of tamoxifen therapy, *n* = 12. Bars represent mean±SEM, *p < 0.05, **p < 0.01.

In breast cancers, there were significantly increased levels of miRNA-29a, miRNA-30c, and miRNA-148a in the cancerous tissue compared with normal adjacent breast tissue, Figure 2. No significant changes were seen in normal breast tissue of these miRNAs during various hormone exposures. Similarly, no changes were seen in plasma samples (data not shown). miRNA-146a did not exhibit any change in cancerous tissue but the levels were significantly decreased in normal breast tissue of postmenopausal women compared with pre-menopausal women, Figure 2. In the luteal phase the levels reached borderline significance with a p-value of 0.07 but no changes were seen after tamoxifen therapy, Figure 2. miRNA-193b, -203, -451, and -452 were unaltered in breast cancers but in normal breast tissue miRNA-193b and miRNA-203 exhibited significantly decreased levels in postmenopausal breast tissue and increased levels in the luteal phase of the menstrual cycle suggesting that hormone exposure may affect their levels, Figure 3.

### NPM1 and Ago2 were detectable in microdialysis samples

To estimate the *in vitro* recovery of the transport proteins i.e. to what degree the proteins pass the membrane, microdialysis catheters were placed in known concentrations of the proteins and perfused under the same conditions as *in vivo.* The concentration of the proteins in the outgoing perfusate was then measured. However, as the *in vitro* recovery will be an estimate of the *in vivo* recovery, as temperature and tissue pressure may be different in *in vivo,* all microdialysis data are presented as raw data. The *in vitro* recovery of NPM1 over a 10 mm microdialysis membrane perfused at 0.5 μl/min was 34±1.6%, *n* = 3 and the level in microdialysates of human tissue was 311±24 pg/ml, *n* = 3. NPM1 was not detectable in plasma samples. The *in vitro* recovery of Ago 2 was 16.6±0.7%, *n* = 3 and the level in microdialysates of human tissue was 87±24 pg/ml, *n* = 3 and in plasma 30±6.5 pg/ml, *n* = 3.

### miRNAs are stable in microdialysates

As the microdialysis membrane prevents larger molecules such as enzymes to diffuse into the sample the degradation of compounds in the microdialysate is very low. To investigate if this was the case also for miRNAs we first explored the extraction variability. As shown in a Supplementary Figure S2A the extraction variability of miRNAs in microdialysates was very low for extracted and pre-amplificated endogenous miRNAs as well as for the spike in control. Next, we validated the stability of miRNAs in microdialysis samples without extraction and pre-amplification. The samples were subjected to 0, 3, and 6 freeze-thaw cycles or incubated at −20°C, +4°C, or +20°C for 24 hours. Despite these different conditions no variability of the expression levels was detected, Supplementary Figure S2B-S2C.

Contrary to the microdialysates, plasma contains all kinds of circulating proteins, enzymes, and cellular contamination that may interfere with the quality of the sample and the performance of all steps in the analytical process of miRNA. In our hands, six separate experiments of all steps in the process of detecting three different miRNA resulted in a few CT values > 40, which is in line with previous data [29]. To correct for these technical variations in miRNA recovery, a spike in control, cel-miRNA-67-3p, was used for normalization of plasma samples as previously described [29].

When RNA is isolated from tissues variable amounts of contaminating cellular DNA may be present in the samples. However, microdialysis samples contain only soluble free compounds from the extracellular space i.e. only free extracellular DNA fragments would have a possibility to the pass the membrane. The microdialysis membrane we used in this study had a molecular weight cut-off value, which prevents the majority of free DNA fragments to pass. This was also verified in our microdialysis samples where no levels of DNA were detected. As there were undetectable levels of DNA in the samples the risk of including false positives in our data was very low.

## DISCUSSION

To the best of our knowledge this is the first study showing tissue specific expression of extracellular miRNAs in human breast cancers and normal human breast tissue *in vivo*. Our data show distinct patterns of extracellular miRNAs in breast cancers of women as well as in normal breast tissue during various hormone exposures.

In breast cancers we found significantly increased levels of extracellular miRNA-21, miRNA- 29a, miRNA-30c, and miRNA-148a. miRNA-21 has been implicated to play a role in cancer progression by affecting the tumor suppressor PTEN and in breast cancer patients miRNA-21 has been found in increased levels in serum [24, 30]. Our data also revealed increased levels of miRNA-21 in normal breast tissue during the luteal phase of the menstrual cycle i.e. during high sex steroid exposure whereas tamoxifen exposure decreased the levels significantly. A hormonal regulation of miRNA-21 in normal breast tissue was further supported by the data of pre- and postmenopausal women as the levels were significantly decreased in the postmenopausal cohort. This seems to be a breast tissue specific change as no differences were found in microdialysates from abdominal subcutaneous fat or in plasma. These results may suggest that miRNA-21 is a possible therapeutic target for breast cancer treatment as well as for sex steroid dependent breast carcinogenesis.

Surprisingly we found significantly increased levels of extracellular miRNA-29a in breast cancers compared with normal adjacent breast tissue. The miRNA-29 family has been associated with tumor suppressing properties including cell cycle regulation, cell migration, and metastasis formation [28]. However, these data refers to intracellular levels, only one study has previously investigated circulating miRNA-29a and found it to be increased in colorectal cancer patients compared with controls [20]. Similar to miRNA-29a cellular expression of miRNA-30c has been associated with decreased metastasis formation in experimental breast cancer and increased progression free survival in in breast cancer patients [19, 31]. No studies have investigated circulating miRNA-30c in breast cancer patients. Regarding miRNA-148a conflicting results in cancer patients have been reported. Recently, high plasma levels of miRNA-148a were reported to be associated with decreased progression free survival in patients with colorectal cancer and multiple myeloma [21, 32]. This is in line with our data showing significantly increased extracellular level in breast cancers compared with normal adjacent breast tissue. However, cellular miRNA-148a has been associated with inhibition of epithelial-mesenchymal transition (EMT) and metastasis formation [33]. Similar to miRNA-148a, the literature regarding the association and thereby the effects of cellular and circulating miRNA-203 differs. Circulating plasma miRNA-203 has been found to be elevated in metastatic breast cancer (MTC) patients with increased numbers of circulating tumor cells (CTC) compared to MTC patients without detectable CTC or controls [23]. However, cellular miRNA-203 may inhibit bone metastasis in prostate cancer in experimental models whereas in breast cancer models the miRNA-200 family has been shown to increase metastasis formation by affecting EMT [26, 34]. In breast cancers the extracellular miRNA-203 levels did not differ significantly from normal adjacent breast tissue but we found significantly increased extracellular levels of miRNA-203 in premenopausal women compared to postmenopausal women and increased levels during the luteal phase of the menstrual cycle suggesting a hormonal effect on the expression of this miRNA although tamoxifen did not affect its levels. Cellular miRNA-146a seems to be up-regulated in metastatic breast cancer cell lines [22] and a very small study has shown increased plasma levels of miRNA-146a in breast cancer patients [35]. We did not find any difference in miRNA-146a levels in breast cancer tissue but the levels in postmenopausal women were lower than in pre-menopausal women suggesting that miRNA-146a may have some physiological function in normal breast tissue. miRNA-193b did not differ in breast cancers but was decreased in postmenopausal women and increased in the luteal phase of the menstrual cycle whereas tamoxifen therapy did not change the levels. There are to our knowledge, no reports in circulating miRNA-193b but in breast cancer, cellular miRNA-193b has been shown to be down-regulated [27]. Regarding miRNA-451 and miRNA-452 no differences were found. Interestingly, previous reports have shown that breast cancer cells releases miRNA-451 whereas non-malignant cells retain this miRNA [25]. However, our data did not show any difference in extracellular levels of miRNA-451 in breast cancer *vs* normal breast tissue.

Despite high activity of RNase in blood circulating miRNAs have been found to be stable suggesting that they are protected from enzyme degradation by different mechanisms including transport of miRNAs in membrane bound vesicles or protein-complexes [4–6]. Vesicles that carry miRNAs include exosomes with a size of 50-90 nm or other membrane bound particles up to 1 μm in diameter [36, 37]. The data generated from microdialysates cannot originate from vesicle bound miRNAs as the pore size of the microdialysis membrane would exclude these compounds. In the present paper we have used a microdialysis membrane with a molecular cutoff of 100 kDa to sample the extracellular miRNAs. The cut-off value is based on a nominal molecular weight cut-off i.e. the ability of the membrane to retain 90% of a globular macromolecule of a defined molecular weight. However, the shape and charge of the molecule, tissue pressure and temperature will affect membrane performance. Recently it has been proposed that the majority of circulating miRNAs are bound to proteins protecting from RNase activities including Ago 2 of approximately 100 kDa, the 37 kDa protein NPM1, or apolipoproteins with molecular sizes allowing for passage over the microdialysis membrane [4, 5, 38]. Although Ago 2 theoretically would have a very low recovery over the microdialysis membrane due to its molecular size we show that the *in vitro* recovery of this protein was approximately 16% and the levels in microdialysis samples from human tissue were in the range of 90 pg/ml, higher than in plasma samples where the levels were approximately 30 pg/ml. NPM1 was readily detected in microdialysates from human tissue with levels of approximately 300 pg/ml and the *in vitro* recovery was 34%. Interestingly, NPM1 was undetectable in plasma. These data suggest that the local tissue microenvironment may contain specific proteins or levels of proteins not reflected in blood samples. This finding further stresses the need of local investigations of extracellular miRNAs and highlights that the origin of miRNAs in plasma or serum never can be a reflection of an individual organ but will rather reflect the total body net amount of extracellular miRNAs. Nonetheless, there may still be some cancer specific miRNAs or panels of miRNAs in blood that may help in discriminating healthy individuals from cancer patients but there are still challenges to overcome before this can be a routine clinical practice.

## CONCLUSION

We show here, for the first time, that miRNAs are readily detectable in microdialysastes from human subjects and exhibit specific patterns of extracellular miRNAs locally in breast cancers and normal breast tissue. We do believe that our findings have significance for the understanding of the role of these extracellular miRNAs in breast cancers and normal breast tissue as no changes were found in extracellular fat tissue or plasma of the specific miRNAs investigated in our study. This emphasizes the need of tissue specific investigations of extracellular miRNAs and our data suggest that several extracellular miRNAs in breast tissue may be regulated by hormone exposure. Further studies will shed light on the specific roles of extracellular miRNAs in breast cancer initiation and progression and investigations of locally released miRNAs will be crucial in this process.

## MATERIALS AND METHODS

### Subjects

The regional ethical review board of Linköping approved the study and all women gave their informed consent. Microdialysis was performed in 33 women. Thirteen women, age 51-86 years, diagnosed with breast cancer were investigated before surgery with microdialysis intratumorally in the breast cancer and in normal adjacent breast tissue. Tumor histology, size, immunohistochemistry of estrogen (ER)- and progesterone (PR) receptors, HER-2 receptor, and Nottingham histological grade (NHG) according to Elston Ellis scoring system were determined at the Department of Pathology and Cytology, University Hospital of Linköping and presented in Table 1.

**Table 1:** Characteristics of patients subjected to intratumoral microdialysis. All patients were postmenopausal One catheter was inserted within the breast cancer and the another catheter was inserted in adjacent normal breast tissue the day before surgery. All cancers were HER-2 negative

Patient	Age	Tumor Size (mm)	Grade (NHG)	ER (%)	PR (%)	Plasma Estradiol (pmol/l)	Plasma Progesterone (nmol/l)
1	51	20	3	>50	0	125	1.0
2	66	20	2	>50	0	30	3.7
3	56	17	2	>50	0	84	2.7
4	57	21	3	>50	0	58	0.7
5	59	10	2	>50	10	57	0.9
6	86	60	3	>50	>50	70	1.0
7	70	22	2	>50	>50	56	2.4
8	52	25	3	>50	10-50	46	0.4
9	67	18	2	>50	>50	23	1.0
10	68	15	2	>50	0	51	1.3
11	65	50	2	>50	0	42	0.6
12	68	13	2	>50	10-50	35	0.8
13	68	24	2	>50	>50	78	1.2

ER=estrogen receptor, PR=progesterone receptor.

Twelve postmenopausal women (age 59-70 years), who had undergone breast surgery for early breast cancer, were subjected to microdialysis in their normal unaffected breast and subcutaneous abdominal fat, before and after six weeks of tamoxifen therapy (20 mg/day) which was prescribed as an adjuvant therapy in routine clinical practice, Table 2. Eight healthy volunteer pre-menopausal women (age 21-30 years) with a history of regular menstrual cycles (cycle length 27-34 days) without any use of sex steroid-containing contraceptive within the past three months were investigated with microdialysis in breast tissue and subcutaneous abdominal fat during the follicular and luteal phases of one menstrual cycle, Table 3. Blood was drawn in EDTA containing tubes from all women on the same days as the microdialysis investigations were performed.

**Table 2:** Characteristics of postmenopausal patients subjected to microdialysis in their normal unaffected breast and in subcutaneous fat at two time points; before and after six weeks of tamoxifen therapy All patients had previously undergone surgery for early breast cancer and tamoxifen was prescribed in clinical routine

Patient	Age	Plasma Estradiol (pmol/l)	Plasma Progesterone (nmol/l)
1	70	59	0.44
2	60	65	1.10
3	63	38	1.10
4	61	35	0.40
5	68	36	1.00
6	59	13	1.20
7	68	35	0.70
8	59	70	0.20
9	66	15	0.10
10	70	105	0.20
11	63	48	0.50
12	65	66	0.44

**Table 3:** Characteristics of premenopausal healthy volunteers subjected to microdialysis in normal breast tissue and in subcutaneous fat at two time points; in the follicular and in the luteal phase of one menstrual cycle

Volunteers	Age	Plasma Estradiol follicular phase (pmol/l)	Plasma Estradiol luteal phase (pmol/l)	Plasma Progesterone follicular phase (nmol/l)	Plasma Progesterone luteal phase (nmol/l)
1	30	95	502	1.3	23
2	21	83	260	1.3	22
3	24	171	280	1.8	32
4	22	89	450	2.7	35
5	24	133	476	2.2	52
6	23	85	370	1.0	25
7	25	102	555	1.1	13
8	30	98	356	1.4	45

### Procedure

Prior to insertion of the microdialysis catheters 0.5 ml lidocain (10 mg*/*ml) was administrated intracutaneously. In women with ongoing breast cancer investigated before surgery one microdialysis catheter was inserted within the cancerous tissue and the other microdialysis catheter was inserted into normal adjacent breast tissue. In women investigated in their normal healthy breast one microdialysis catheter was placed in the upper lateral quadrant of the breast and directed towards the nipple as previously described [39–42] and one in abdominal subcutaneous fat. In the premenopausal women the catheters were inserted in the left breast and in the postmenopausal women starting tamoxifen their unaffected healthy breast was subjected to microdialysis. Microdialysis catheters (CMA 71*/*Microdialysis AB, Solna, Sweden), which consists of a tubular dialysis membrane (0.52 mm in diameter, 100,000 atomic mass cut-off) glued to the end of a double-lumen tube (80 mm long x 0.8 mm in diameter), were inserted via a splitable introducer (CMA Microdialysis AB), connected to a microinfusion pump (CMA 107, CMA Microdialysis AB) and perfused with NaCl 154 mmol*/*L and hydroxyethyl starch 60 g/l (Voluven^®^, Fresenius Kabi, Uppsala, Sweden), at a perfusion rate of 0.5 μl/min. In the women investigated in their normal breast a 20 mm long microdialysis membrane was used whereas in the women investigated within their breast cancer and normal adjacent breast tissue a 10 mm long membrane was used. After a 60-min equilibration period, the outgoing perfusate was stored at −70°C for subsequent analysis. EDTA plasma was collected at the time for microdialysis and stored at −70°C.

### miRNA extraction

miRNA extraction was performed using the ZR RNA MicroPrep kit from Zymo Research (Zymo Research Corporation, CA, USA). 30 μl microdialysate or 100 μl plasma were used for extraction and miRNAs were eluted in 6 μl or 8 μl nuclease-free water respectively. In plasma samples a spike in control, the synthetic nucleotide Caenorhabditis elegans miRNA-67-3p (cel-miRNA-67-3p), was added together with the plasma sample in the lysis buffer prior extraction. Levels of DNA in the samples were analyzed using NanoDrop spectrophotometer.

### Reverse transcription and pre-amplification of miRNAs

TaqMan MicroRNA Reverse Transcription Kit (Applied Biosystems, Darmstadt, Germany) was used for reverse transcription (RT). The 7.5 μl-reverse transcription reaction contained 0.8 μl Megaplex RT primers, 0.2 μl 100 mM dNTP, 1.5 μl Multiscribe Reverse Transcriptase, 50 U/μl, 0.8 μl RT-buffer, 0.9 μl MgCl_2_ (25 mM), 0.1 μl Rnase inhibitor, 0.2 μl Nuclease free water and 3 μl extracted miRNA sample. Pre-amplification of converted DNA (cDNA) was performed with TaqMan PreAmp Master Mix (2X) (Applied Biosystems) and Megaplex PreAmp Primers (10X) (Applied Biosystems) using the Gene Amp PCR System 9700. The reaction volume of 25 μl consisted of: 12.5 μl TaqMan PreAmp Master Mix, 2.5 μl Megaplex PreAmp Primers, 7.5 μl nuclease-free water and 2.5 μl of the reverse transcript product. The pre-amplification was run on Gene Amp PCR System 9700.

### miRNA assays

Following the pre-amplification step miRNA profiling was performed using TaqMan^®^ Array human microRNA A card v2.1 (Applied Biosystems), enabling quantification of 377 human miRNAs. Briefly, 9 μl of the diluted pre-amplificated product was mixed with 450 μl TaqMan^®^ Universal Master Mix II, No UNG (Applied Biosystems) and 441 μl nuclease-free water. 100 μl of the PCR reaction mix was dispensed into each port of the TaqMan MicroRNA array card. The cards were centrifuged and sealed. RT-PCR was performed on a 7900HT Thermal cycler. Data was analyzed using SDS Relative Quantification Mananger Software version 1.2.1 (Applied BioSystems). miRNAs with a detectable transcription were defined as those with a cycle threshold (C_T_) less than 35. Individual target miRNAs were assayed in a 15 μl PCR reaction mix containing 7.5 μl TaqMan Universal PCR Mastermix No Amperase UNG 2X (AppliedBiosystems), 5.75 μl nuclease free water, 0.75 μl TaqMan microRNA assay 20X (AppliedBiosystems) and 1 μl of the pre-amplificated c-DNA product assayed using the 7900HT Thermal cycler.

### Absolute quantification of miRNAs by real-time PCR

Synthetic miRNAs (miRNA mimic) purchased from Ambion representing the mature miRNA sequences (miRBase v.20), specified in Supplementary Table S2, were used as standards for absolute quantification. The synthetic oligonucleotides were treated the same way as microdialysis samples, except for the extraction step, and then serial diluted with nuclease free water to generate standard curves for all miRNA assays. A line was fit from each dilution using C_T_ values within the linear range for concentration calculations. Absolute concentration of miRNA was converted to fmol/l.

### Variability and stability test for miRNAs in microdialysis samples

Pooled microdialysis samples were divided in six 30 μl portions to test the extraction variation of miRNAs. Cel-mir-67-3p was added to the sample as a spike in control. Each sample was extracted separately using the ZR RNA MicroPrep kit from Zymo Research and eluted in 6 μl portions. 3 μl of the eluted miRNAs were converted to c-DNA, pre-amplificated, and then analyzed with RT-PCR. The microdialysis samples were subjected to 0, 3, and 6 freeze-thaw cycles and were maintained at −20°C, +4°C, and +20°C for 24 hours. Without any miRNA extraction, reverse transcription was performed on all samples directly after 24 hours. The endogenous miR-16 was quantified using RT-PCR for C_T_ value comparison.

### Detection of nucleophosmin 1 and argonaute 2 protein

Microdialysates and plasma were analyzed using Nucleophosmin1 (NPM1) ELISA kit (Cusabio Biotech Co., LTD, Wuhan, China) and argonaute 2 protein (Ago 2) ELISA kit (antibodies-online inc. Atlanta, US).

### Statistical analyses

Statistical analyses were performed using paired Wilcoxon matched-pairs signed rank test and unpaired Mann-Whitney U test where appropriate. All tests were two-sided and a *p* < 0.05 was considered as statistically significant.

## SUPPLEMENTARY MATERIAL TABLES AND FIGURES


