# Supplementary material for: Tissue specific expression of extracellular microRNA in human breast cancers and normal human breast tissue in vivo
Source: Oncotarget. 2015 May 8;6(26):22959–69. doi: 10.18632/oncotarget.4038 (PMC4673212; doi:10.18632/oncotarget.4038)
Supplement: Supplementary file 1 [file oncotarget-06-22959-s001.pdf]

Tissue specific expression of extracellular microRNA in human breast cancers and normal human breast tissue *in vivo*

Supplementary Material

Table S1: Detectable extracellular miRNAs with a CT below 35 from qRT-PCR profiling of microdialysis from human breast cancer

Detector	CT	Detector	CT	Detector	CT
hsa-let-7b-5p	25.670	hsa-miR-143-3p	27.706	hsa-miR-345-5p	31.709
hsa-let-7c-5p	30.088	hsa-miR-145-5p	27.830	hsa-miR-363-3p	33.137
hsa-let-7e-5p	33.663	hsa-miR-146a-5p	25.432	hsa-miR-365a-3p	30.452
hsa-miR-10a-5p	30.135	hsa-miR-146b-5p	26.519	hsa-miR-367-3p	27.342
hsa-miR-16-5p	22.343	hsa-miR-148a-3p	30.831	hsa-miR-374a-5p	30.419
hsa-miR-17-5p	25.889	hsa-miR-150-5p	29.918	hsa-miR-375	30.543
hsa-miR-19b-3p	24.124	hsa-miR-152-3p	29.380	hsa-miR-376c-3p	31.503
hsa-miR-19a-3p	27.414	hsa-miR-155	24.783	hsa-miR-376a-3p	34.019
hsa-miR-20a-5p	26.045	hsa-miR-185-5p	30.664	hsa-miR-423-5p	33.528
hsa-miR-20b-5p	28.889	hsa-miR-186-5p	25.967	hsa-miR-425-5p	25.299
hsa-miR-21-5p	26.151	hsa-miR-187-3p	28.944	hsa-miR-449b-5p	34.145
hsa-miR-24-3p	25.278	hsa-miR-191-5p	27.863	hsa-miR-451a	25.836
hsa-miR-25-3p	28.784	hsa-miR-192-5p	28.139	hsa-miR-452-5p	31.622
hsa-miR-26b-5p	29.165	hsa-miR-193b-3p	26.270	hsa-miR-454-3p	31.690
hsa-miR-26a-5p	31.482	hsa-miR-193a-5p	31.811	hsa-miR-455-3p	34.938
hsa-miR-27a-3p	29.698	hsa-miR-194-5p	33.885	hsa-miR-483-5p	28.365
hsa-miR-27b-3p	32.516	hsa-miR-195-5p	26.680	hsa-miR-484	25.017
hsa-miR-28-3p	30.561	hsa-miR-196b-5p	31.835	hsa-miR-485-5p	24.716
hsa-miR-29a-3p	27.108	hsa-miR-197-3p	27.480	hsa-miR-491-5p	33.427
hsa-miR-29c-3p	29.790	hsa-miR-199a-3p	27.101	hsa-miR-511-5p	32.151
hsa-miR-30c-5p	28.624	hsa-miR-200c-3p	27.912	hsa-miR-518f-3p	31.570
hsa-miR-30b-5p	34.148	hsa-miR-200b-3p	30.379	hsa-miR-523-3p	27.686
hsa-miR-34a-5p	30.267	hsa-miR-200a-3p	32.255	hsa-miR-532-5p	30.350
hsa-miR-92a-3p	26.810	hsa-miR-202-3p	29.982	hsa-miR-532-3p	32.176
hsa-miR-93-5p	28.241	hsa-miR-203a	31.259	hsa-miR-539-5p	33.631
hsa-miR-96-5p	31.244	hsa-miR-204-5p	34.861	hsa-miR-548a-3p	34.355
hsa-miR-99a-5p	30.178	hsa-miR-208-3p	31.515	hsa-miR-574-3p	26.514
hsa-miR-100-5p	28.615	hsa-miR-208b-3p	34.706	hsa-miR-590-5p	29.065
hsa-miR-101-3p	33.727	hsa-miR-214-3p	28.951	hsa-miR-598-3p	33.063
hsa-miR-106a-5p	26.006	hsa-miR-218-5p	29.025	hsa-miR-618	31.111
hsa-miR-106b-5p	29.439	hsa-miR-221-3p	31.273	hsa-miR-628-5p	23.304
hsa-miR-125b-5p	27.584	hsa-miR-222-3p	24.622	hsa-miR-636	31.253
hsa-miR-126-3p	25.243	hsa-miR-223-3p	21.520	hsa-miR-660-5p	34.957
hsa-miR-130a-3p	31.267	hsa-miR-224-5p	29.369	hsa-miR-708-5p	31.845
hsa-miR-130b-3p	34.310	hsa-miR-302a-3p	26.717	hsa-miR-875-3p	30.544
hsa-miR-132-3p	29.075	hsa-miR-320a	25.454	hsa-miR-886-5p	33.881
hsa-miR-133a-3p	33.064	hsa-miR-324-3p	31.647		

hsa-miR-134-5p	31.360	hsa-miR-328-3p	30.215
hsa-miR-139-5p	31.625	hsa-miR-331-3p	32.004
hsa-miR-140-5p	28.831	hsa-miR-331-5p	32.125
hsa-miR-140-3p	31.062	hsa-miR-335-5p	30.169
hsa-miR-141-3p	32.085	hsa-miR-339-3p	32.826
hsa-miR-142-3p	31.409	hsa-miR-340-5p	32.048
hsa-miR-142-5p	31.802	hsa-miR-342-3p	27.565

Table S2: Mature miRNAs assayed on human microdialysates and plasma samples mapped to miRBase version V20

Mature ID	Mature miRNA Sequence
hsa-miR-16-5p	UAGCAGCACGUAAAUAUUGGCG
hsa-miR-19b-3p	UGUGCAAAUCCAUGCAAAACUGA
hsa-miR-21-5p	UAGCUUAUCAGACUGAUGUUGA
hsa-miR-29a-3p	UAGCACCAUCUGAAAUCGGUUA
hsa-miR-30c-5p	UGUAAACAUCCUACACUCUCAGC
hsa-miR-146a-5p	UGAGAACUGAAUUCCAUGGGUU
hsa-miR-148a-3p	UCAGUGCACUACAGAACUUUGU
hsa-miR-193b	AACUGGCCCUCAAAGUCCCGCU
hsa-miR-203a	GUGAAAUGUUUAGGACCACUAG
hsa-miR-451a	AAACCGUUACCAUUACUGAGUU
hsa-miR-452-5p	AACUGUUUGCAGAGGAAACUGA
cel-miR-67-3p	UCACAACCUCCUAGAAAGAGUAGA

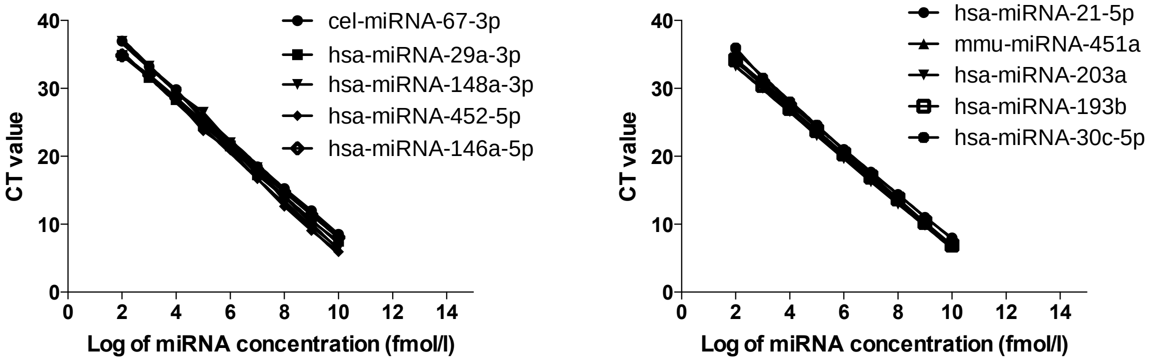

Figure S1: ***Standard curves for quantification of human miRNAs.***

Standard curves were generated for every miRNA by using a dilution of a known concentration of synthetic miRNAs. The real-time PCR showed a broad dynamic range with a correlation coefficient ranging from 0.9982 to 0.9998.

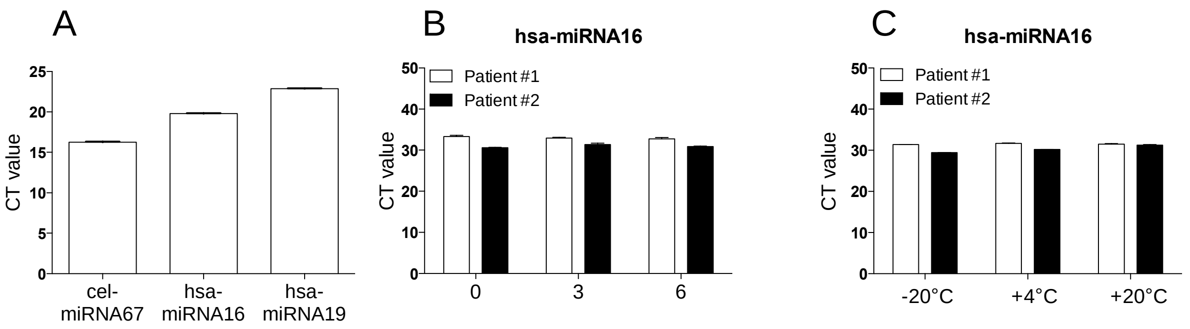

Figure S2: *High stability and low extraction variability of microRNAs in microdialysis samples.*

A. Extraction variability of miRNAs in microdialysates of extracted and pre-amplificated endogenous miRNAs and spike in control, n=6 in each group.

B. Stability of miRNAs without extraction and pre-amplification in microdialysis samples subjected to 0, 3, and 6 freeze-thaw cycles, n=3 in each group.

C. Stability of miRNAs without extraction and pre-amplification in microdialysis samples subjected to -20°C, +4°C or +20°C for 24 hours, n=3 in each group.
